# Impact of Very Hot Drink Consumption Habits, Age, and Sex, on Taste Sensitivity

**DOI:** 10.3390/foods10051139

**Published:** 2021-05-20

**Authors:** Christophe MARTIN, Eric NEYRAUD

**Affiliations:** 1 Centre des Sciences du Goût et de l’Alimentation, AgroSup Dijon, CNRS, INRAE, Université Bourgogne-Franche-Comté, F-21000 Dijon, France; eric.neyraud@inrae.fr; 2 PROBE Research Infrastructure, Chemosens Facility, F-21000 Dijon, France

**Keywords:** taste sensitivity, temperature, hot drink, age, gender, T@sty

## Abstract

The temperature range for consuming hot drinks includes temperatures that can damage cells on the tongue. We hypothesized that the consumption of very hot drinks can lead to a decrease in the ability to perceive low concentrations of tastants. We evaluated the ability to perceive low concentrations of five prototypical sapid compounds in 42 women and 40 men aged 18–65. A questionnaire made it possible to collect the usual frequencies (number of unit/day) and consumption temperature levels (medium hot/very hot) for four very common hot drinks (coffee, tea, herbal infusions, and hot chocolate). Our results showed that subjects who consumed very hot drinks (versus medium hot) were less sensitive to sweet (*p* = 0.020) and salty (*p* = 0.046) tastes. An aggravating effect of high consumption frequencies was only shown for sweet taste (*p* = 0.036). Moreover, our data also showed that women were more sensitive than men to sour, bitter, and umami tastes (*p* values < 0.05), as well as that taste sensitivity decreases with age, especially after 50 years old (all tastes; *p* values < 0.05). These findings strengthen our knowledge about the influence of sex and age on taste sensitivity, and they provide knowledge on the influence of consumption habits related to hot drinks on taste sensitivity.

## 1. Introduction

Coffee and tea are the two most frequently consumed hot drinks in the world [1]. Other widely consumed hot drinks include herbal infusions, hot chocolate, and mate. These drinks are obtained by solid–liquid extraction (coffee, tea, mate, etc.) or by dissolution using a solvent (example: hot chocolate and instant coffee). In any case, their preparation involves the use of a solvent that is heated to a very high temperature, most often water. For example, brewing coffee requires water with a temperature ideally between 85 and 95 °C [2]. At the end of the preparation, the temperature of the drink is lower than that of the given solvent. However, the temperature remains very high. In the catering industry, the recommended temperature for keeping drinks warm is between 85 and 88 °C [2]. According to Brown and Diller [3], hot beverages such as tea, hot chocolate, and coffee are frequently served at temperatures between 71.1 and 85 °C. These results were corroborated by data from a lawsuit against a fast food restaurant chain in the United States that showed that coffee that caused burns was dispensed at a temperature between 75 and 88 °C [4]. Furthermore, Verst et al. [4] showed that the average serving temperature of coffee approximately two minutes after preparation varied between 66 and 77 °C depending on the given machine and the context (household or food service industry). Hot drinks are not always consumed directly after preparation. The time between the preparation and the introduction of hot drinks into the mouth allows for cooling. This drop in temperature depends on several factors such as the initial temperature of the drink, the time between preparation and its introduction into the mouth, the thermal properties of the container, the ambient temperature, and the amount of other added substances such as milk and cream. [3]. When consumers deem the temperature satisfactory, the hot drinks are taken into the mouth and consumed. In practice, this consumption is sometimes preceded by a test phase intended to estimate the risk of burns. Several studies have made it possible to determine the temperature that is considered to be ideal by consumers. For coffee, which has been frequently studied, the ideal temperature seems to be between 60 and 70 °C [2,4,5,6]. For tea or mate, the preferred temperature seems to be close to 70 °C [6]. However, the temperature that is considered ideal varies across consumers. In fact, in a cohort study involving more than 40,000 regular tea drinkers, Islami et al. [7] showed that 39.0% of participants drank their tea at a temperature below 60 °C, 38.9% drank it between 60 and 64 °C, and 22.0% drank it at more than 65 °C.

The introduction of hot drinks into the mouth causes an increase in the temperature in the tissues of the tongue, the oral cavity, and then the esophagus. The temperature increase at the surface of the tissues primarily depends on the temperature of the liquid, contact time, and the thermal conductivity of the exposed surfaces. Lee et al. [8] showed that coffee at a temperature of 60 °C could increase the surface area temperature of the tongue to 53 °C. This temperature is above the threshold for the emergence of pain on the tongue surface, which is approximately 46–48 °C [6,8]. The fact that the preferred temperature exceeds the threshold temperature for pain could be explained by the short residence time of the hot drink and by the phenomenon of habituation [6,8,9]. To our knowledge, there have been no experimental studies concerning burns on the surface of the tongue caused by heat from liquids in the mouth. However, there are data on the epidermis from other parts of the body. According to Borchgrevink et al. [2], 45 °C is the equilibrium point of the skin. Below this exposure temperature, there is no damage. Between 45 and 51 °C, the rate of cell destruction doubles for every degree of Celsius. Between 60 and 65 °C, the destruction rate is 10 million times higher than it would be at a temperature of 45 °C. Above 70 °C, the destruction of the cells is complete, even for contact times of less than 1 s [6]. In addition, a mathematical model that accounted for the specific characteristics of the external tissues of the tongue (mucosa) was developed by Brown and Diller [3] with the objective of simulating burns and quantifying the extent of thermal damage. When applied to drinking coffee, this model indicated that a temperature of 57.8 °C limits the risk of burns while maintaining consumer satisfaction.

The usual temperatures for serving and consuming hot drinks are therefore within a temperature range liable to cause cellular damage in the oral cavity, including on the tongue. After examining more than 1000 observational and experimental studies, a working group from the International Agency for Research on Cancer (IARC) came to the conclusion that drinking very hot beverages at temperatures above 65 °C is probably carcinogenic to humans [1,10]. It was also specified that it is the temperature of the liquid and not the nature of the liquid that is in question. Taken together, these findings indicate that very hot drinks can constitute, to some extent, a physical aggression on the cells of the oral cavity, including the tongue.

It is well-known that taste is fundamentally important for food selection. Thus, it appears essential that the taste buds function normally in order to properly perceive this characteristic of foods. Therefore, we thought it would be interesting to know whether hot drink consumption could have an impact on taste sensitivity. Surprisingly, no study has yet addressed this topic. Nevertheless, the papillae containing the taste buds are primarily expressed on the dorsal surface of the tongue [11], especially on the anterior two-thirds of the tongue [12]. This position makes the papillae particularly vulnerable since the tip of the tongue is, along with the lips, the first part of the body that is in direct contact with hot drinks. This part of the tongue is also the one that is most often involved in taste dysfunctions [12]. The life span of human taste buds is approximately ten days, with a range of 3–30 days. Normally, taste buds appear to degenerate and regenerate at the same rate [13]. However, we wonder if the aggression represented by regular exposure to very hot drinks could influence their renewal rate or more generally disrupt their normal functioning and simultaneously reduce the ability to perceive tastes. In this case, the taste capacities of consumers who were accustomed to drinking very hot beverages should be lower than that of consumers accustomed to lower temperatures.

Among the factors having an impact on taste sensitivity, apart from illnesses or medical treatment, are gender and age. The literature regarding differences in sensitivity between men and women has been contradictory. Some studies have shown a greater taste sensitivity for women [12,14,15], while other studies have shown no significant difference [16]. Additional data are needed on the subject. The effect of age is much clearer. Two comprehensive literature reviews clearly showed that taste abilities decline with age [16,17]. In any event, there is a clear consensus on the need to consider these factors when assessing taste sensitivity.

This article presents the results of a study conducted to assess the possible negative influence of consuming very hot drinks on the ability to perceive tastes. Our hypothesis was that subjects who drink very hot liquids would exhibit a lower taste sensitivity than subjects who drink liquids at lower temperatures. We also hypothesized that a high frequency of consumption would be an aggravating factor. The aim of the study was to evaluate the taste sensitivity of a group of 82 subjects and to characterize their hot drink consumption habits. We then studied the links between these two datasets. We focused our attention on the most frequently consumed drink. In fact, due to the high frequency of the consumption of this drink, it constitutes the bulk of the risk associated with high temperatures. We considered age and sex of participants in the analyses as factors of variation due to their recognized influence on taste sensitivity [16,17].

## 2. Materials and Methods

### 2.1. Subjects

The 82 subjects who participated in this study were all part of a larger panel recruited for a research project called Taste and Oral Microbiota (TOM). They were all normal-weight nonsmokers, did not follow any long-term drug treatment (lasting more than one month), had not taken antibiotics or antiseptic mouthwashes, and had received no dental care in the 30 days preceding the experiment. In addition, for the purposes of the present study, they all consumed at least one of the following hot drinks: coffee, tea, herbal infusions, or hot chocolate (at least 2 times/week). The panel was composed of 42 women and 40 men aged 18–65 (mean: 42; median: 43 years old). The sex ratios (number of men/number of women) for the 18–35, 36–50, and 51–65 age groups were 0.8, 1.0, and 1.1, respectively. All the subjects signed a consent form and were compensated with up to 30€ for their participation. A competent ethics committee approved the protocol for the taste sensitivity assessment (Comité de Protection des Personnes Ouest V, n ° 2016-A01954-47).

### 2.2. Study Design

**Figure 1:**
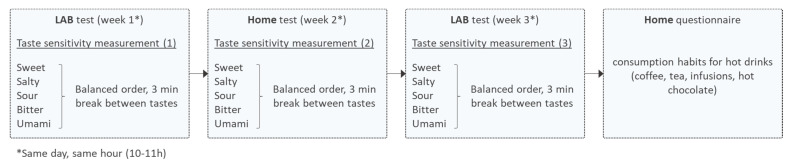
General design of the study.

Figure 1 shows the general design of the study. For each taste, three measurements were performed with a one-week interval, always between 10 and 11 a.m. During one session, all five tastes were tested. The order of evaluation for the different tastes varied according to Williams’ Latin square. The first and third taste sensitivity measurements took place in our laboratory in a room dedicated to the study of oral physiology. These sessions were individual and always performed by the same investigator. The stimulated saliva flow was measured during these two laboratory sessions. The second measure of taste sensitivity was performed at home, during the second week of the study, without the presence of the investigator. The material needed for the test (see Section 2.3.1) was given to the subjects at the end of the first laboratory session. Responses to the sensitivity test were given via an internet questionnaire. The objective of this measurement performed at home was to assess the ability to perform such measures outside the laboratory without making people move in the future. Finally, the subjects completed an internet questionnaire on hot drink consumption habits after the end of the sensitivity measurements.

### 2.3. Taste Sensitivity Test

For the taste sensitivity evaluation, we chose to focus on the ability to perceive low concentrations close to the detection threshold. This type of measurement is very often used in clinical studies to detect taste dysfunctions [18]. The sensitivity test used in this study made it possible to specifically stimulate the first third of the tongue (regional test). This type of test was suitable for our purpose because this part of the tongue is the first to come into contact with hot liquids when they are introduced into the mouth. In addition, this area is the most involved in taste dysfunction [12].

We assessed the sensitivity for the five basic tastes. Fructose for sweet taste (CAS: 57-48-7, Cooper, France), sodium chloride for salty taste (NaCl, CAS: 7647-14-5, Cooper, France), citric acid for sour taste (CAS: 5949-29-1, Cooper, France), quinine hydrochloride for bitter taste (Quinine HCl, CAS: 6119-47-7, Merck KGaA, Germany), and monosodium glutamate for umami taste (MSG, CAS: 6106-04-3, Merck KGaA, Germany) were used as the prototypical taste compounds. These five molecules were all of food or pharmacopoeia qualities. We chose fructose for technical reasons (the viscosity of the solutions to be printed; see Section 2.3.1).

#### 2.3.1. Material

**Figure 2:**
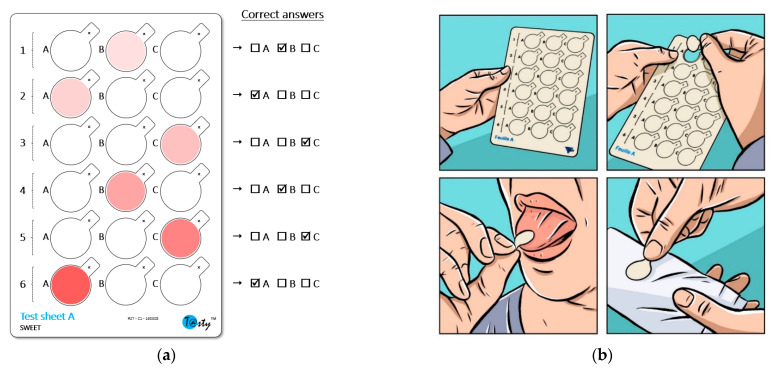
(**a**) An example of a test sheet (allowing for one sensitivity measurement to a given taste). The color symbolizes the location of the tasty discs and the increasing intensity of the taste intensity. Dimensions of the sheet: 11 × 16 × 0.06 cm. Discs: 18 mm in diameter and 2.54 cm^2^ in area. (**b**) The procedure for detaching and tasting the discs was as follows: detach the disc from the test sheet by holding it by the gripping tab, put the upper face of the disc (marked with a small cross) in contact with the tip of the tongue (first third) for a maximum of 4–5 s, and then discard the disc. After a short pause (10 s), subjects could evaluate the next triplet of discs.

The sensitivity test used here, called T@sty, was in the form of several edible test sheets, each allowing for one sensitivity measurement to a given taste. Each test sheet consisted of six triplets of detachable precut discs to be placed in contact with the tongue (Figure 2a). The triplets all consisted of one tasty disc and two neutral discs. The tastants, which were in a solution of deionized water, were printed on the surface of the discs (wafer paper) using a food-grade inkjet printer. The position of the tasty disc varied randomly depending on the triplets and the test sheets. The surface concentration of the tastant and the intensity of taste gradually increased from the first to the sixth triplets. Distilled water was printed in the same manner on the neutral discs to make them look the same as the tasty discs. Several pretests made it possible to determine the appropriate concentrations leading to a discriminating test. The pretests consisted of carrying out several preliminary studies involving 60–200 subjects and observing the distribution of the scores obtained. The concentrations were adjusted until a distribution close to a Gaussian distribution was obtained. The final surface concentrations were as follows: 2.1, 2.7, 4.1, 7.1, 16.1, and 35.4 µg/cm^2^ (fructose); 4.9, 5.2, 5.6, 6.7, 8.8, and 12.6 µg/cm^2^ (NaCl); 3.6, 4.1, 5.6, 9.5, 17.1, and 24.7 µg/cm^2^ (citric acid); 2.9, 9.5, 19.2, 48.8, 98.3, and 133.5 ng/cm^2^ (quinine HCl); and 1.1, 1.6, 3.79, 19.6, and 29.6 µg/cm^2^ (MSG).

#### 2.3.2. Test Principle

For each test sheet, and thus for each taste, the subjects had to successively evaluate the six triplets, starting with the triplet located at the top of the sheet and then moving downwards. The lowest concentration was used for the triplet numbered 1, and the highest concentration was used for the triplet numbered 6. For example, we used the following concentrations for the tasty disc for fructose triplets 1–6, respectively: 2.1, 2.7, 4.1, 7.1, 16.1, and 35.4 µg/cm^2^. For each triplet, the subjects had to successively taste the three discs, from left to right, and then indicate the tasty disc (forced choice). The name of the tested taste was indicated on the support. The subjects therefore knew which taste was being tested. However, because some weakly concentrated tastants were known to sometimes have a different taste quality than expected at higher concentrations, the subjects were informed that if there was any doubt about the perceived sensation, they should find the disc that was different from the other two. Figure 2b shows how to taste a disc. The evaluation took less than five minutes per taste sheet. Rinsing with water followed by a 3-min break (return to normal salivation) was imposed between the different test sheets dedicated to the different tastes. Training samples were used during the first session to familiarize the subjects with the procedure and the stimulus to be perceived (consisting of a reduced test sheet with a single triplet of discs). The maximum surface concentration of the range was used for the tasty disc of this triplet.

#### 2.3.3. Sensitivity Score

**Figure 3:**
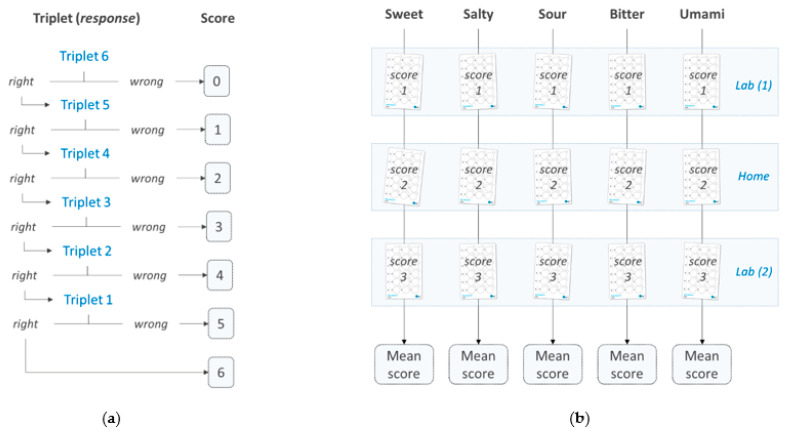
(**a**) Determination of the sensitivity score (for a given test sheet). The score was based on the greatest sequence of correct answers, starting from the sixth triplet of discs (highest concentration). (**b**) For each taste, three measurements were performed, each giving rise to a sensitivity score. The average of the three scores was used to determine the sensitivity of the subjects.

Figure 3a schematizes the rule for determining the sensitivity scores. This approach was based on the best estimate threshold (BET) method described in standard E679-19 [19], with the difference that the result of the test was not expressed as a concentration but as a sensitivity score ranging from 0 to 6. The higher the score was, the more the given subject exhibited a good ability to detect low concentrations of the given tastant. The average of the three replicates was used to determine the sensitivity of the subjects (Figure 3b).

### 2.4. Questionnaire on Hot Drink Consumption Habits

The participants completed an internet questionnaire regarding their hot drink consumption habits. The objective of this questionnaire was to determine, for each subject, the most frequently consumed drink (named “favorite hot drink” in this document) and its usual temperature level at consumption.

The four hot drinks selected for this study (coffee, tea, herbal infusions, and hot chocolate) were the most frequently consumed in France during the winter (the period studied for the survey). For each drink, the subjects were asked to specify (i) the frequency of consumption (number of units/cups per day, week or month) and (ii) the usual temperature of consumption (lukewarm, medium hot, very hot, or boiling). The participants were informed that they had to answer all the questions by considering the last four months as a reference period (i.e., from December to March—the winter season). The questions were asked with the following phrasing: (i) During the last four months, how often did you drink each of the drinks below (coffee, tea, herbal infusions, and hot chocolate)? (ii) At what temperature do you prefer to consume the drinks below (coffee, tea, herbal infusions, and hot chocolate)?

### 2.5. Salivary Flow

Unstimulated whole saliva was collected by passive drooling into a pre-weighed tube [20]. This measurement was performed before the taste sensitivity measurement during the two laboratory sessions. Salivary flow was measured as a possible confounder impacting on the primary outcome variable.

### 2.6. Data Analysis

The data were analyzed with the XLSTAT software (Addinsoft (2020), XLSTAT statistical and data analysis solution, Paris, France. https://www.xlstat.com, 2021).

#### 2.6.1. Validity of Sensitivity Measurements

The repeatability of the measurements was evaluated by calculating, for each taste, the Pearson correlation coefficients between the sensitivity scores obtained during the three replicates. A significant linear relationship was expected. In addition, for each taste, an ANOVA (type III) was performed to determine whether the scores obtained during the three measurements differed (model: sensitivity score = subject + replicate + error). Tukey’s post-hoc test (significance level set at 0.05) made it possible to identify which replicates differed. For each taste and subject, the average of the three replicates was used for the following analyses.

#### 2.6.2. Interindividual Variability

For each taste, descriptive statistics linked to the distributions of the mean scores made it possible to comment on the interindividual variability.

#### 2.6.3. Link between Sensitivity for the Five Tastes

The Pearson correlation coefficient was used to study the correlations between the scores obtained for the different tastes. A principal component analysis (PCA; biplot representation), which was performed on the correlation matrix, provided a multidimensional representation of the links between the scores obtained for the different tastes.

#### 2.6.4. Hot Drink Consumption Habits

Descriptive statistics were used to comment on the global hot drink consumption patterns (the frequency of consumption and usual temperature level). The Chi^2^ test was used to compare the frequency of consumption for men and women and for the different age groups in the study.

#### 2.6.5. Influence of Hot Drink Consumption, Age and Sex on Taste Sensitivity

For each taste, a covariance analysis (ANCOVA, type III) was performed with the objective of studying the influence of the consumption frequency (number of units per month) and usual temperature (medium hot or very hot) on taste sensitivity while accounting for participant gender (male/female) and age group (18–25, 26–50, or 51–65 years old). The model was as follows: sensitivity score = temperature level + age group + sex + consumption frequency + error). A 5% threshold was chosen to test the significance of the factors. We considered that *p* values between 0.05 and 0.01 reflected a trend. For each of the factors, Tukey’s post-hoc test (with the significance level set at 0.05) was used for multiple comparisons of the means. The normalized coefficient from the ANCOVA was used to clarify the direction of the relationship between the consumption frequencies and sensitivity scores.

#### 2.6.6. Salivary Flow

For each subject, the values collected during the two replicates were averaged. The links between the mean flows and the sensitivity scores were investigated using the Pearson correlation coefficient. An ANOVA (type III) was performed to find whether there were significant differences in salivation between men and women on the one hand and between age groups on the other hand (model: flow = age group + sex + error).

## 3. Results

### 3.1. Taste Sensitivity

#### 3.1.1. Validity of Taste Sensitivity Measurements

For each taste, the Pearson correlation coefficient was calculated between each replicate (replicates 1–2, 2–3, and 1–3). The averages of the three coefficients were as follows: r(82) = 0.53 (sweet); r(82) = 0.40 (salty); r(82) = 0.46 (sour); r(82) = 0.49 (bitter); and r(82) = 0.47 (umami). The details on the correlations are presented in Table 1. Overall, the highest correlation coefficient was observed for consecutive sessions (replicates 1–2 and 2–3) and the lowest was for sessions spaced 2 weeks apart (replicates 1–3). The results of the ANOVAs indicated that the scores varied from one replicate to another only for sweet (F(2, 162) = 4.14; *p* = 0.018) and salty (F(2, 162) = 6.55; *p* = 0.002) tastes (Table 2). In both cases, the scores obtained during the first measurement were higher than those during the second measurement (0.6–0.7 points on a scale of 0–6). The scores obtained during the second and third measurements did not significantly differ. No significant difference was found between the three measures for sour (F(2, 162) = 1.70; *p* = 0.19), bitter (F(2, 162) = 0.23; *p* = 0.79), and umami (F(2, 162) = 0.23; *p* = 0.80) tastes. Overall, the context of the measurement (lab versus home) did not seem to have influenced the resulting scores. In fact, out of 10 possible comparisons (lab/home), only two significant differences were observed (Table 2, Tukey’s test).

#### 3.1.2. Interindividual Variability

**Figure 4:**
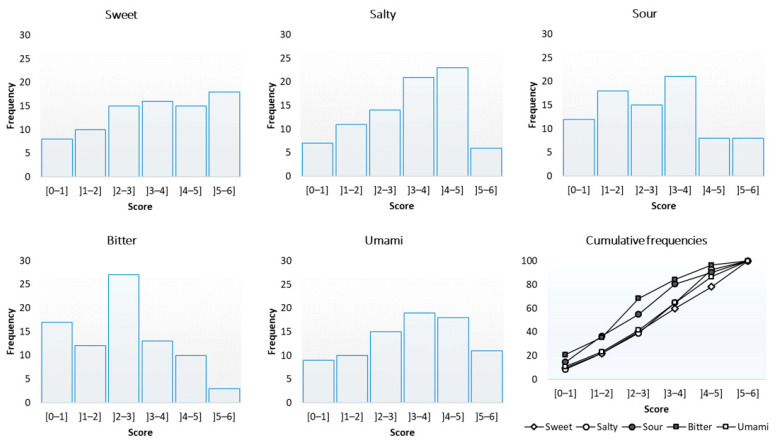
Distribution of taste sensitivity scores (82 subjects).

Figure 4 shows the distribution of the sensitivity scores for the five tastes. Overall, the distributions showed a high interindividual variability in sensitivity levels, regardless of the taste. The medians of the distributions were as follows: 3.7 (sweet), 3.7 (salty), 2.8 (sour), 2.7 (bitter), and 3.7 (umami).

#### 3.1.3. Sensitivity for the Five Tastes

**Figure 5:**
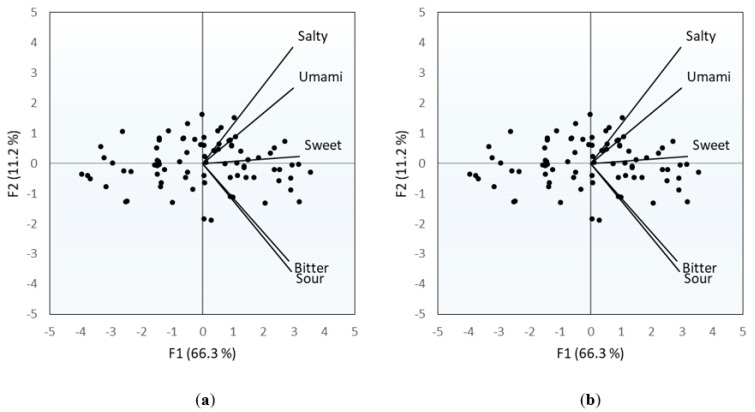
Links between sensitivity for the five tastes (82 subjects). PCA (correlation): axes 1–2 (**a**) and 1–3 (**b**).

The Pearson’s correlation coefficients indicated a significant correlation between scores obtained for the different tastes (*p* < 0.0001 for all correlations), with coefficients ranging from 0.49 to 0.66. A comparison of the average correlation coefficients (the averages for the coefficients calculated between each taste and the four others) revealed that the bitter taste was, on average, the least correlated with the others (r(82) = 0.42; *p* < 0.0001). Sweet taste was, on average, the most correlated with the others (r(82) = 0.63; *p* < 0.0001). Figure 5 summarizes the correlations between the sensitivities for the different tastes. For both PCA representations (F1–F2 and F1–F3), the point clouds that represent the 82 subjects are stretched along axis 1. This axis, which explains approximately 66% of the observed variability, seems to reflect a general sensitivity. Subjects for whom the mark is on the left side of the charts were generally less sensitive to taste than subjects whose mark is on the right side. The orientation and the direction of the vectors representing the tastes give some indications about the degree of connection between the scores obtained for the different tastes. The vector representing the sweet taste is always well-correlated with axis 1, which suggests that this taste best reflects the general sensitivity of a subject.

### 3.2. Hot Drink Consumption Habits

#### 3.2.1. Most Frequently Consumed Hot Drink (Favorite Hot Drink)

**Figure 6:**
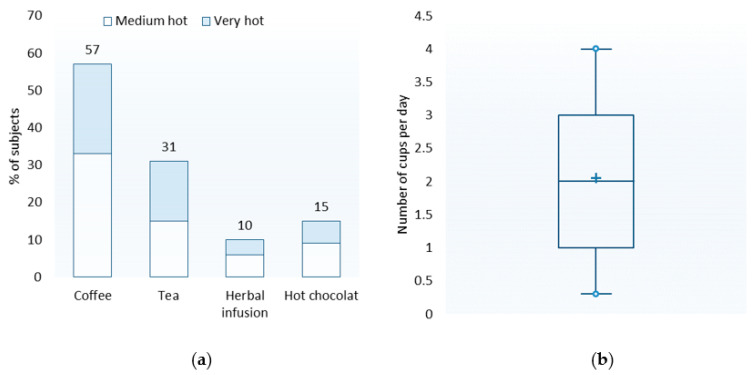
Favorite drink. (**a**) Percentage of subjects per drink and usual temperature level of consumption. (**b**) Distribution of consumption frequencies for the favorite drink.

The reported frequency of consumption made it possible to determine the favorite hot drink of each subject. For 57% of the subjects, coffee was the favorite hot drink. The percentages for the other drinks were as follows: 31% (tea), 10% (herbal infusion), and 15% (hot chocolate) (Figure 6a). The sum of these percentages exceeds 100, because for nine of the 82 participants, several hot drinks were consumed with the same frequency. In this case, we kept only the drink consumed at the highest temperature level for the analyses to follow. In fact, this drink was found to constitute the bulk of the risk associated with high temperatures. Tea was more often the favorite hot drink for women than for men (CHI^2^ (1; *n* = 82) = 19.47; *p* < 0.0001). There were no significant differences between men and women for other drinks. Furthermore, coffee was more often the favorite hot drink of 51–65-year-olds than 18–35-year-olds (CHI^2^ (2; *n* = 82) = 15.79; *p* = 0.0002). Conversely, hot chocolate was more often the favorite hot drink of 18–35-year-olds than 51–65-year-olds (CHI^2^ (2; *n* = 82) = 9.63; *p* = 0.005).

#### 3.2.2. Usual Consumption Temperature

The terms “lukewarm” and “boiling” were not used to qualify the usual temperature level for consuming hot drinks (favorite drink). Figure 6a shows the percentage of subjects consuming their favorite drink at the medium hot and very hot levels. Overall, 56.1% and 43.9% of subjects reported drinking their favorite drinks at the medium hot and very hot levels, respectively. 

#### 3.2.3. Frequency of Consumption (Favorite Drink)

Figure 6b shows the distribution of the consumption frequencies for the favorite drink. The mean and the median were equal to approximately two cups per day. The minimum and maximum frequencies recorded here were 0.3 and 4.0 cups per day, respectively (i.e., 9–120 cups per month).

### 3.3. Salivary Flow

Salivary flow was not correlated with sensitivity scores for any of the five tastes, with r(82) = 0.01 and *p* = 0.958 (sweet); r(82) = 0.14 and *p* = 0.201 (salty); r(82) = −0.02 and *p* = 0.831 (sour); r(82) = 0.06 and *p* = 0.567 (bitter); and r(82) = 0.06 and *p* = 0.590 (umami). Notably, no differences in salivary flow were observed between either men and women (F(1, 81) = 1.66; *p* = 0.201) or the different age classes (F(1, 81) = 0.16; *p* = 0.851).

### 3.4. Influence of Hot Drink Consumption Habits, Age, and Sex on Taste Sensitivity

#### 3.4.1. Influence of the Usual Temperature Level

**Figure 7:**
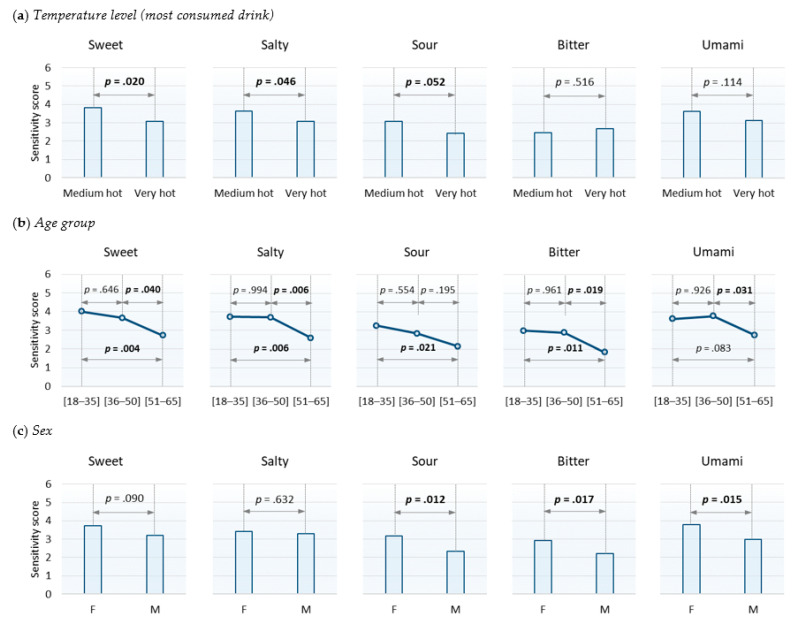
Summary of multiple comparisons (Tukey HSD; significance level set at 5%). Means and *p* values associated with Tukey’s results. The *p* values in bold are less than or equal to 0.05.

The ANCOVA performed for each taste (Table 3) showed that the sensitivity scores depended on the usual temperature level for the sweet (F(1, 76) = 5.62; *p* = 0.020) and salty (F(1, 76) = 4.11; *p* = 0.046) tastes. The same trend was observed for the sour taste (F(1, 76) = 3.90; *p* = 0.052). Multiple comparisons of means indicated that subjects for whom the usual temperature level was very hot had, on average, lower sensitivity scores than subjects whose usual temperature level was medium hot (Figure 7a). This effect was not significant for the bitter (F(1, 76) = 0.43; *p* = 0.516) and umami tastes (F(1, 76) = 2.56; *p* = 0.114).

#### 3.4.2. Influence of the Consumption Frequency

The frequency of consumption had an influence on the sensitivity scores for the sweet taste (F(1, 76) = 4.56; *p* = 0.036). The normalized coefficient from the ANCOVA indicated that the lower the frequency of consumption was, the higher the sweet taste sensitivity score. The influence of the consumption frequency was not significant for the other tastes (Table 3).

#### 3.4.3. Influence of the Age Group

The ANCOVA showed that the sensitivity scores depended on the age of the participants. All tastes were impacted (Table 3). Overall, multiple comparisons of means showed a decrease in sensitivity scores with increasing age. The differences between the sensitivity scores of 18–35-year-olds and 51–65-year-olds were significant for sweet (F(2, 76) = 5.92; *p* = 0.004), salty (F(2, 76) = 6.74; *p* = 0.002), sour (F(2, 76) = 3.83; *p* = 0.026), bitter (F(2, 76) = 5.49; *p* = 0.006), and umami (F(2, 76) = 3.85; *p* = 0.026) tastes. The differences between the sensitivity scores of 36–50-year-olds and 51–65-year-olds were significant for all the tastes except for the sour taste. The sensitivity scores of 18–35 and 36–50-year-olds did not significantly differ for any of the five tastes (Figure 7b).

#### 3.4.4. Influence of Sex

The ANCOVA showed that the sensitivity scores depended on the sex of the participants for sour (F(1, 76) = 6.67; *p* = 0.012), bitter (F(1, 76) = 5.92; *p* = 0.017), and umami (F(1, 76) = 6.20; *p* = 0.015) tastes (Table 3). The same tendency was observed for the sweet taste (F(1.76) = 2.96; *p* = 0.090). Multiple comparisons of means showed that women obtained higher sensitivity scores than men (Figure 7c). The sex effect was not decisive for the salty taste (F(1, 76) = 0.23; *p* = 0.632).

## 4. Discussion

### 4.1. Taste Sensitivity

The sensitivity test used for this study is relatively new. Only one published study presented data collected using this test [20]. It is therefore necessary to provide some elements showing the validity of the measurement by comparing with other well-established methods. In a study by Mueller et al. [21], the Pearson correlation coefficients calculated between scores obtained 12 days apart and with 69 subjects were 0.43 (sweet), 0.34 (salty), 0.40 (sour), and 0.56 (bitter) for the taste strips and 0.50 (sweet), 0.37 (salty), 0.36 (sour), and 0.61 (bitter) for the well-established three-drop-method [22]. The average correlation coefficients observed from one repetition to another with the T@sty test were within the same order of magnitude (0.40–0.53). We consider this result to be satisfactory, especially since one of the three replicates was performed at home. Higher correlation coefficients can be found in the literature, in particular for the three-drop method, but with delays of a few tens of minutes between the replicates [23]. For sweet and salty tastes only, the scores obtained during the first measurement were higher than they were during the second measurement. No other difference was found between the replicates. The use of a training sample may have resulted in slightly better scores, at least for the sweet and salty tastes. It would be interesting to generalize the use of this sample and not to restrict its use to the first measurement. We also observed that the scores obtained during the home assessment were generally not different from the scores obtained in the laboratory. This finding suggests that the test used here is suitable for nonlaboratory use. Apart from our hypotheses, we have shown that the salivary flow was not linked to the sensitivity scores obtained for the different tastes. The dry support used to produce the test was therefore not a limitation in our case.

We observed a strong interindividual variability in the sensitivity levels for the five tastes. This strong variability was widely accepted by the scientific community [16,17,18]. Moreover, our results support the idea of a generalized taste sensitivity, which has also been suggested by the results of other studies [24,25,26]. In other words, when a subject exhibited a satisfactory sensitivity for one taste, they often also had a satisfactory sensitivity for other tastes. However, this statement must be qualified by specifying that the correlation coefficients between the tastes ranged from 0.49 to 0.66, which corresponded to the coefficients of determination (R^2^) that ranged from 0.24 to 0.44. Therefore, the quality of the prediction was not perfect. Our data indicated that the sweet taste seemed to correlate the most, on average, with other tastes. Therefore, this taste seems to be the most representative of general sensitivity. If only one taste had to be selected to estimate overall sensitivity, then the sweet taste seems to be the best candidate. Remember, however, that the prediction power was still quite low (R^2^ = 0.44 on average).

### 4.2. Consumption Frequency and the Usual Temperature of Hot Drinks

The consumption frequencies collected for the favorite drink averaged 2.0 units per day, with a range of 0.3–4 units per day. These data are credible. Indeed, according to the results of the INCA 3 study [27], the average consumption of hot drinks in France is 268.9 g per day or 2.15 units (cups) per day if we consider that one medium-sized cup contains approximately 125 g of hot drink. The differences in consumption observed between men and women and between age groups also confirmed the value of considering these two variables during the analyses.

To qualify the usual temperature level of hot drink consumption (favorite drink), participants only used the terms medium hot and very hot. The percentages of subjects for these two temperature levels were relatively balanced. However, the medium hot level was strongest for hot chocolate. The addition of milk, which is quite common for this type of hot drink, may explain this result. Indeed, adding substances such as milk and cream ultimately lead to a reduction in consumption temperature [3,4,6]. We also observed that the frequency of subjects consuming their tea at a very hot level was high compared to other drinks. This observation was consistent with the literature, indicating that the consumer’s preferred drinking temperature for tea and mate is close to 70 °C [6]. These observations do not provide any new information about hot drink consumption habits, but they were quite reassuring about the quality of the data collected here.

### 4.3. Influence of Hot Drink Consumption, Age, and Sex on Taste Sensitivity

In accordance with our hypotheses, we focused our attention on the influence of the most frequently consumed hot drink (usual temperature level and frequency of consumption). The sex and age group of the participants were taken into account in the analyses.

#### 4.3.1. Influence of the Usual Temperature Level

For the sweet and salty tastes, the sensitivity scores of subjects who consumed their hot drink at a very hot level were significantly lower than those of subjects who consumed their drink at a medium hot level. This observation suggested the possible deleterious effect of high temperatures on the ability to perceive these two tastes. The same tendency was observed for the sour taste. However, this effect was not found for bitter and umami tastes. These last two tastes are known to depend on a genetic polymorphism that explains the inability of a small proportion of the population to properly perceive the tastes of certain bitter or umami molecules [28,29,30,31]. Therefore, it is possible that, in some cases, the low sensitivity scores obtained for these two tastes was due to this genetic inability rather than to their drinking habits for hot drinks. In this situation, these particular cases would have degraded the relationship that we expected. In fact, subjects who do not have suitable receptors for bitter or umami molecules cannot satisfactorily perceive the corresponding tastes, regardless of their hot drink consumption habits. In any case, the results partially validated our hypothesis on the possible deleterious effect of high temperatures linked to the consumption of very hot drinks.

#### 4.3.2. Influence of the Consumption Frequency

We hypothesized that a high consumption frequency would be an aggravating factor in the deleterious effects of high temperatures. The results only partially confirmed this hypothesis. In fact, there was only a significant effect for the sweet taste, and the *p* values observed for the other tastes were far from the significance threshold. These results suggested that the frequency of hot drink consumption generally has little or no significant effect on the ability to perceive tastes. However, the effect of the consumption frequency may not be linear. In this case, the consumption frequencies of our panel, although presenting a certain variability, were perhaps globally above a threshold beyond which the variations in consumption frequency no longer had any influence on taste sensitivity. It should be noted that our panel was composed of regular consumers of hot drinks. A complementary study comparing non-consumers, occasional consumers, and regular consumers of hot drinks would perhaps more clearly show a consumption frequency effect.

#### 4.3.3. Influence of Age

Our study showed that the ability to perceive low concentrations of fructose, sodium chloride, citric acid, quinine hydrochloride, and monosodium glutamate decreases with increasing age. Literature on the impact of age on taste abilities is abundant. Booth et al. [13] and Methven et al. [17] performed very comprehensive reviews on the subject. Comparisons of the results of the various studies cited in these reviews reveal some contradictory results, but, overall, a decline in taste capacity with increasing age was often highlighted. Most of the apparent contradictions highlighted by these literature reviews can probably be explained by differences in the selected methodologies. Concerning the detection thresholds, the authors of [17] showed that a very large majority of the 23 studies included in the meta-analysis concluded that the detection thresholds increased with age (70% of studies for bitter and sweet tastes, 80% for salty and sour tastes, and 100% studies for umami taste (two studies only)). Our results were consistent with this general observation. According to Methven et al. [17] and Mojet et al. [16], a decline in tasting capacity would not occur until after the age of 60 years. Our results showed that it could happen a little sooner than that. In fact, our results showed that a decline seemed to begin after 35 years and become significant after 50 years. Even if the reduction in salivary flow is sometimes a reason given to explain the lower sensitivity observed in older people [32], in our case, we can exclude this possibility because the salivary flows observed for the different age groups were not significantly different. Moreover, the subjects involved in our study were not “very old,” since they were at most 65 years old.

#### 4.3.4. Influence of Sex

We showed that women’s sensitivity scores were, on average, significantly higher than that of men for the sour, bitter, and umami tastes. The same trend was observed for the sweet taste. However, we did not find any significant difference for the salty taste. The literature on the differences in taste sensitivity between males and females has revealed seemingly contradictory results. These contradictions very clearly appeared in a paper by Mojet et al. [16], in which the conclusions of several studies were listed. It is therefore possible to find references in a similar vein to our results, as well as also references that are in disagreement. The diversity of the studied populations, used approaches, and used tests to estimate taste sensitivity probably explain a large part of these contradictions. We can still say that, according to the literature, when a difference has been noted between the two sexes, it has almost always been in favor of a greater sensitivity in women [12,16,32,33,34,35,36]. The results of our study support this general observation about differences in taste sensitivity between men and women.

## 5. Strengths and Limitations

To our knowledge, this is the first time that the link between the consumption of very hot drinks and the ability to perceive tastes has been studied. The hypothesis that the consumption of very hot drinks could have a deleterious effect on taste sensitivity was raised from the fact, both simple and strong, that hot drinks are sometimes taken into the mouth at temperatures likely to cause cell damage on the tongue.

For this study, which should be considered a first approach that was intended to give an initial insight into the strength of the studied factors, we chose to evaluate the temperature perceived during the consumption of hot drinks using a questionnaire (self-reported temperature level). The primary reason that guided this choice was the cumbersome nature of the protocols implemented to obtain a reliable objective/instrumental measurement of the consumption temperature [2,6,8], which was accentuated by the number of drinks we targeted (four drinks) and the number of participants involved in our study (*n* = 82). For a preliminary test, we preferred the subjective measurement presented in this document.

The risk associated with using self-reported temperature levels is that among subjects who reported drinking “very hot” there were likely many subjects who actually drink their hot drinks at very high temperatures. However, a few subjects who are used to drinking at lower temperatures may have responded “very hot” because they have a high temperature sensitivity or low tolerance to high temperatures. We could have the opposite reasoning for the subjects who declared drinking “medium hot.” However, we believe that this potential bias related to the subjective perception of temperature or tolerance for high temperature is likely to reduce the significance of the links observed between the consumption temperature and sensitivity for sweet and salty tastes—not exacerbate it. Indeed, if our hypothesis concerning the deleterious effect of high temperatures is correct, then the situation that would maximize the significance of this effect would correspond to the case where all the subjects who drink at high/low temperatures, respectively, answer “very hot”/“medium hot.” This is the reason that we are confident about the observed effects, even though we believe that further experimentation is necessary to fully verify these preliminary results.

Several other pieces of evidence suggest that a self-reported temperature level measurement makes sense, although it is not as accurate as an instrumental measurement. First, an IARC report specified that drinking temperature can be considered “hot” between 50 and 65 °C and “very hot” at temperatures above 65 °C [1,10]. This indicates that these two terms used in everyday language cover a physical reality. Second, the attribution of a term, for example “very hot,” to qualify the perceived temperature of a food results from a lesson partly based on collective experience, i.e., on the opinions expressed by other people about shared experiences. The use of these terms is therefore probably relatively homogeneous. Moreover, a study conducted by Dirler et al. [6] showed that, on average, coffee was perceived as “too hot” for temperatures beyond 66 °C, with a standard deviation of 3 °C. This very small standard deviation suggests that the judgments made by the subjects regarding the temperature of their drinks are relatively homogeneous.

Despite these arguments, it would be interesting to extend this study into a similar work based on the instrumental evaluation on the temperatures of hot drinks. These measurements could relate to all the hot drinks consumed to account for a possible additional effect of occasionally consumed drinks. The study could even be extended to solid foods. Indeed, solid foods can also occasionally be vectors of high temperatures. For example, a study by Lachenmeier and Lachenmeier [37] showed that a slice of 2.5-mm-thick boiled potato with a temperature of 70 °C could cause the tongue temperature to rise to almost 55 °C. Physiological measurements, in particular those intended to compare the state of the papillae and taste buds in consumers of very hot versus moderately hot foods, would provide a better understanding of the origin of the effects observed in this study. Finally, the influence of texture sensitivity on hot drink consumption habits (usual temperature) and the influence of thermal status on taste perception thresholds could be interesting factors to study in future work.

## 6. Conclusions

Our results complement current knowledge regarding the effect of age and sex on taste sensitivity. This study also provided new knowledge suggesting that the very high temperatures delivered by hot drinks could have a negative effect on the ability to perceive low concentrations of certain tastes. These results constitute a first contribution to the study of high temperatures as a source of variation in taste sensitivity. Other experiments must be performed to confirm the observed effects and to understand the mechanisms underlying the deleterious effect of very hot drinks on taste sensitivity.

## Figures and Tables

**Table 1:** Repeatability of the taste sensitivity test. Pearson correlation coefficients calculated between the scores obtained during the three repetitions.

Taste	Pearson Correlation Coefficient—r(82), *p* Value			
	Replicate 1 vs. 2	Replicate 2 vs. 3	Replicate 1 vs. 3	Mean (3 Replicates)
Sweet	0.54; *p* < 0.0001	0.57; *p* < 0.0001	0.48; *p* < 0.0001	0.53
Salty	0.47; *p* < 0.0001	0.37; *p* = 0.001	0.35; *p* = 0.001	0.40
Sour	0.51; *p* < 0.0001	0.53; *p* < 0.0001	0.33; *p* = 0.002	0.46
Bitter	0.48; *p* < 0.0001	0.46; *p* < 0.0001	0.53; *p* < 0.0001	0.49
Umami	0.53; *p* < 0.0001	0.39; *p* < 0.0001	0.48; *p* = 0.0003	0.47

**Table 2:** Repeatability of measurements: ANOVA (type III; one analysis by taste); model: score = age group + sex + temperature level + frequency. *p* values: * *p* ≤ 0.05; ** *p* ≤ 0.01. Values with the same letter are not significantly different (Tukey’s test, significance level set at 0.05).

Factor		Sweet	Salty	Sour	Bitter	Umami
Subject	F	4.37	2.9	3.53	3.88	3.56
	DDL	81	81	81	81	81
	*p* value	<0.0001	<0.0001	<0.0001	<0.0001	<0.0001
Replicate	F	4.14	6.55	1.7	0.23	0.23
	DDL	2	2	2	2	2
	*p* value	0.018 **	0.002 *	0.19	0.79	0.80
Tukey’s test		Sweet	Salty	Sour	Bitter	Umami
Replicate 1 (lab)	mean	3.82 a	3.87 a	3.07 a	2.59 a	3.52 a
Replicate 2 (home)	mean	3.23 b	3.15 b	2.68 a	2.67 a	3.43 a
Replicate 3 (lab)	mean	3.57 ab	3.24 b	2.74 a	2.54 a	3.38 a

**Table 3:** Summary of the ANCOVA (type III) carried out (one analysis by taste). Model: score = age group + sex + temperature level + frequency. *p* values smaller than 0.1 are in bold. *p* values: (*) *p* < 0.10, * *p* < 0.05, and ** *p* < 0.01.

Factor	F/*p* Value	Sweet	Salty	Sour	Bitter	Umami
Temperature level	F	5.62	4.11	3.90	0.43	2.56
	*p* value	0.020 *	0.046 *	0.052 (*)	0.516	0.114
Frequency	F	4.56	0.11	1.70	0.06	2.03
	*p* value	0.036 *	0.74	0.197	0.813	0.158
Age group	F	5.92	6.74	3.83	5.49	3.85
	*p* value	0.004 **	0.002 **	0.026 *	0.006 **	0.026 *
Sex	F	2.96	0.23	6.67	5.92	6.20
	*p* value	0.090 (*)	0.632	0.012 *	0.017 *	0.015 *

## Data Availability

The data presented in this study are available on request from the corresponding author.
